# Efficacy and safety of micro-implant anchorage in Angle class II malocclusion orthodontic treatment: A protocol for systematic review and meta-analysis

**DOI:** 10.1097/MD.0000000000023221

**Published:** 2020-12-11

**Authors:** Kaiting Wang, Hongliang Fan, Hongmei Yang, Jianbin Li, Weihong Xie

**Affiliations:** a Department of Medicine, Henan Medical College; b Henan Borui Dental Clinic; c Department of Stomatolagy, The First Affiliated Hospital of Zhengzhou University, Zhengzhou, Henan Province, China.

**Keywords:** micro-implant anchorage, Angle class II malocclusion orthodontic, protocol, systematic review

## Abstract

**Background:**

Angle class II malocclusion is clinically complex and common malocclusion type, which affects beauty. Conventional treatment has the disadvantages of long course of treatment, high cost, easy recurrence and limited curative effect. Clinical practice shows that micro-implant anchorage has certain advantages in the treatment of Angle II malocclusion, but lacks the evidence of evidence-based medicine. This study systematically evaluates the efficacy and safety of micro-implant anchorage in the treatment of Angle class II malocclusion.

**Methods:**

A systematic search was performed by retrieving on English databases (PubMed, Embase, Web of Science, and the Cochrane Library) and Chinese databases (CNKI, Wanfang, Weipu [VIP], CBM). Besides, manually search for Google and Baidu academic of micro-implant anchorage in the treatment of Angle class II malocclusion in randomized controlled clinical research. The retrieval time limit was from the establishment of the database to September 2020. Two researchers independently extracted and evaluated the quality of the data in the included study. A meta-analysis was performed using RevMan5.3 software.

**Results:**

In this study, the efficacy and safety of micro-implant anchorage against Angle class II malocclusion were evaluated by SNA, BNA, ANB, NLA°, Adverse reaction.

**Conclusions:**

This study will provide reliable evidence-based evidence for the clinical application of micro-implant anchorage in the treatment of Angle class II malocclusion.

**Ethics and dissemination:**

Private information from individuals will not be published. This systematic review also does not involve endangering participant rights. Ethical approval was not required. The results may be published in a peer-reviewed journal or disseminated at relevant conferences.

**OSF Registration number:** DOI 10.17605/OSF.IO/UPBR8.

## 1 Introduction

The World Health Organization estimates that malocclusions are the third largest oral health problem after tooth decay and periodontal disease.^[1]^ Angle introduced his famous malocclusion taxonomy in 1899,^[2]^ and Angle II class malocclusion is one of the common type. The clinical incidence is relatively high, the highest among the white race,^[3]^ and the incidence rate in China is about 20.05%.^[4]^ The main clinical manifestations are anterior deep overjet, excessive tooth display, maxillary protrusion, and mandibular retrusion.^[5]^ Because of the disturbance caused by its convex face on patients’ mental health and interpersonal communication, Angle class II malocclusion affects their daily life,^[6,7]^ the deep coverage of anterior teeth also causes periodontal trauma, tooth wear, masticatory difficulty, temporomandibular joint dysfunction, and other adverse effects.^[4]^ Therefore, the patients’ desire for correction is extremely strong, and many Class II patients even choose the second treatment because they are not satisfied with the effect of the previous one. The purpose of clinical correction of the disease is to improve the oral function and profile by correcting the relative relationship between the upper and lower jaw and reducing the coverage of the anterior teeth,^[8]^ mainly through the straight wire appliance (MBT) combined with anchorage design to achieve this goal.^[9]^

In the process of adduction of anterior teeth, it is often necessary to increase the anchorage of posterior teeth in order to increase the adduction of anterior teeth. The traditional anchorage methods include extraoral arch, transverse palatal arch, and the combination of extraoral arch and transverse palatal arch.^[10]^ However, these methods often lead to poor anchorage effect because of poor comfort, poor cooperation of patients and other reasons, it has been reported that the loss of traditional extra-oral strong anchorage can reach 1.6 to 4 mm.^[12]^ Micro-implant anchorage is favored by more and more clinicians and patients because of its small size, flexible implantation site, simple and comfortable operation, no patient cooperation, good stability and reliable anchorage effect. At present, a number of randomized controlled trials have confirmed that micro-implant anchorage has the advantages of shorter course of treatment and better improvement of facial shape and dental arch convexity than traditional anchorage in the correction of Angle Class II malocclusion.^[9,15,16]^ However, there are differences in the research scheme and curative effect of each clinical trial, which leads to the uneven results. Therefore, this study will systematically evaluate the efficacy and safety of micro-implant anchorage and provide evidence-based basis for the clinical application of micro-implant anchorage in the treatment of Angle class II malocclusion.

## 2 Methods

### 2.1 Protocol register

This protocol of systematic review and meta-analysis has been drafted under the guidance of the preferred reporting items for systematic reviews and meta-analyses protocols (PRISMA-P). Moreover, it has been registered on open science framework (OSF) on October 11, 2020 (Registration number: DOI 10.17605/OSF.IO/UPBR8).

### 2.2 Ethics

Since this is a protocol with no patient recruitment and personal information collection, the approval of the ethics committee is not required.

### 2.3 Eligibility criteria

#### 2.3.1 Types of studies

We will collect randomized controlled trials on the treatment of Angle class II malocclusion with micro-implant anchorage, regardless of blinding, publication status, region, but Language will be restricted to Chinese and English.

#### 2.3.2 Object of studies

Patients with Angle Class II malocclusion were diagnosed according to the lateral cephalogram analysis and model analysis (the classification standard was Angle classification^[2]^). There were no restrictions on Chinese nationality, race, age, sex, course of disease, and so on.

#### 2.3.3 Types of interventions

The treatment group: use micro-implant anchorage.

The control group: use traditional anchorage such as extraoral arch, transverse palatal arch, Nance arch and so on.

#### 2.3.4 Types of outcome indicators

1. Primary outcome:
  (a) sella-nasion-Apoint, SNA;
  (b) sella-nasion-Bpoint, BNA;
  (c) Apoint -nasion-Bpoint, ANB.
2. Secondary outcomes:
  (a) NLA°;
  (b) G-Sn-Pg°; UL-E Plane, mm;
  (c) SN-MP°;
  (d) Orthodontic time;
  (e) Adverse reaction.

### 2.4 Exclusion criteria

1. Studies with non-randomized controlled trial or published repeatedly;
2. Studies whose literature are abstract or data are incomplete, or where there are obvious errors that cannot be handled after contacting the author;
3. Studies with high bias risk assessed by randomization or allocation concealment^[17]^;
  Studies which randomizes or allocates concealment as assessed as a high risk of bias
4. Studies which includes patients with periodontal disease or surgical correction;
5. Studies with no relevant literature on outcome indicators.

### 2.5 Search strategy

“Micro-implant anchorage ” (wei xing zhong zhi ti zhi kang), “Angle class II” (an shi II lei) were used for retrieval in Chinese databases, including CNKI, Wanfang Data Knowledge Service Platform, VIP Information Chinese Journal Service Platform, and China Biomedical Database. English retrieval words such as “mini implants,” “micro screws,” “Angle Class II,” “Malocclusion, Angle Class II, Division 1” were used for retrieval in English databases, including PubMed, EMBASE, Web of Science, and the Cochrane Library. In addition, manual retrieval was performed in Baidu and Google academic. The retrieval time was from the establishment of the database to September 2020, and all the domestic and foreign literatures about Mini-implant anchorage for the treatment of Angle class II malocclusion were collected. Take PubMed as an example, and the retrieval strategy is shown in Table 1.

**Table 1:** Search strategy in PubMed database.

Number	Search terms
#1	Mini implants [Title/Abstract]
#2	Micro screws [Title/Abstract]
#3	Micro implants [Title/Abstract]
#4	Miniplate [Title/Abstract]
#5	#1 OR #2 OR#3 OR#4
#6	Angle Class II [MeSH]
#7	Malocclusion, Angle Class II, Division 1 [Title/Abstract]
#8	Angle Class II, Division 1 [Title/Abstract]
#9	Class II Malocclusion, Division 1 [Title/Abstract]
#10	Malocclusion, Angle Class II, Division 2 [Title/Abstract]
#11	Class II Malocclusion, Division 2 [Title/Abstract]
#12	Angle Class II, Division 2 [Title/Abstract]
#13	#6 OR #7 OR #8 OR #9 OR #10 OR #11 OR #12
#14	#5 AND #13

### 2.6 Data screening and extraction

**Figure 1:**
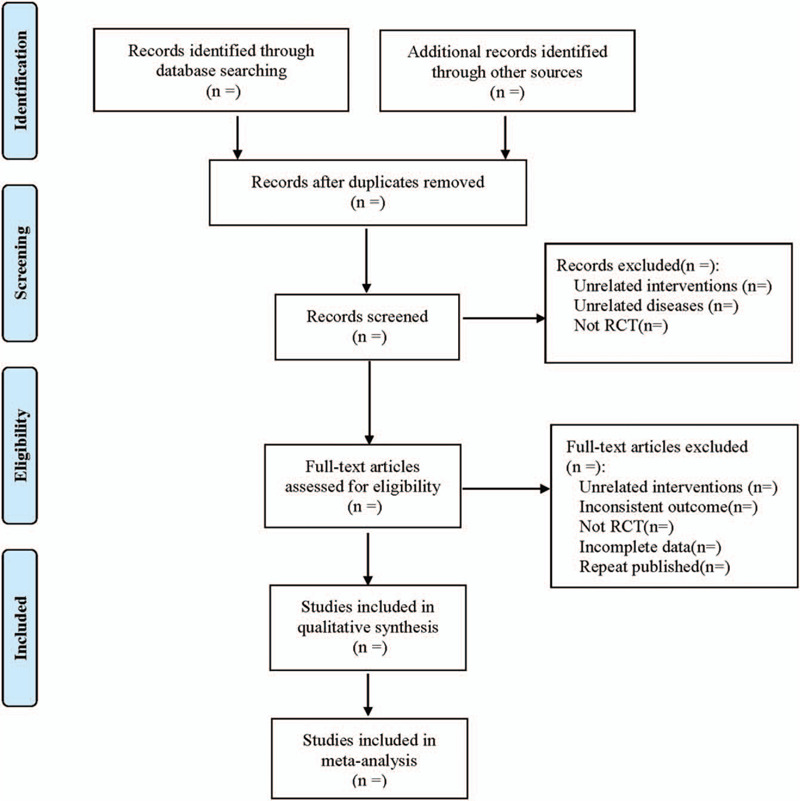
Flow diagram.

Referring to the method of research selection in version 5.0 of the Cochrane collaboration Network system Evaluator Manual, according to the PRISMA flow chart, the two researchers used the EndNote X9 document management software to independently screen and check the literature according to the above inclusion and exclusion criteria, and check each other, if there were different opinions, negotiate with a third party to resolve the differences. At the same time, Excel 2013 was used to extract relevant information, including:

1. Clinical research (title, first author, publication year, and month, sample size, sex ratio, average age, average course of disease);
2. Intervention measures: implant type, implant quantity, implant location, orthodontic time;
3. Evaluation factors of risk bias in randomized controlled studies;
4. Outcome index. The literature screening process is shown in Figure 1.

### 2.7 Literature quality assessment

The Cochrane collaboration's tool for assessing risk of bias was used to assess the risk bias in the included studies. Two researchers determined the literatures from three levels, including low-risk, unclear, and high-risk based on the performance of the included literature in the above evaluation items. After completion, they would recheck. In case of a disagreement, they would discuss. If no agreement could be reached, a decision would be made in consultation with researchers from the third party.

### 2.8 Statistical analysis

#### 2.8.1 Data analysis and processing

The RevMan 5.3 software provided by the Cochrane Collaboration was used for statistical analysis. (1) For dichotomous variables, relative risk (RR) was used for statistics. For continuous variables, Weighted Mean Difference (WMD) was selected when the tools and units of measurement indicators are the same, Standardized Mean Difference (SMD) was selected with different tools or units of measurement, and all the above were represented by effect value and 95% confidence interval (CI). Heterogeneity was determined by χ^2^ and *I*^2^ values. If (*P* ≥ .1, *I*^2^ ≤ 50%), there was low inter-study heterogeneity, and the fixed-effect model was adopted to conduct a meta-analysis. If (*P* < .1, *I*^2^ > 50%), it indicated inter-study heterogeneity and should explore the source of heterogeneity. Analyze and deal with clinical heterogeneity through subgroup analysis. If there was no obvious clinical or methodological heterogeneity, it would be considered as statistical heterogeneity, and the random-effect model would be used for analysis. Descriptive analysis was used if there was significant clinical heterogeneity between the two groups.

#### 2.8.2 Dealing with missing data

If there is missing data in the article, contact the author via email for additional information. If the author cannot be contacted, or the author has lost relevant data, descriptive analysis will be conducted instead of meta-analysis.

#### 2.8.3 Subgroup analysis

Subgroup analysis was carried out according to the age of the patient, which can be divided into two subgroups: children and adults. Subgroup analysis was carried out according to course of treatment; subgroup analysis was carried out according to different types of anchorage used in the control group.

#### 2.8.4 Sensitivity analysis

In order to test the stability of meta-analysis results of indicators, a one-by-one elimination method will be adopted for sensitivity analysis.

#### 2.8.5 Assessment of reporting biases

Funnel plots were used to assess publication bias if no fewer than 10 studies were included in an outcome measure. Moreover, Egger's and Begg's test were used for the evaluation of potential publication bias.

#### 2.8.6 Evidence quality evaluation

The Grading of Recommendations Assessment, Development, and Evaluation (GRADE) will be used to assess the quality of evidence. It contains 5 domains (bias risk, consistency, directness, precision, and publication bias). And the quality of evidence will be rated as high, moderate, low, and very low.

## 3 Discussion

Class II malocclusions represent a significant percentage of orthodontic cases treated in clinical practice. Genetic, environmental, and ethnic factors are the main factors causing this malformation.^[18]^ In the clinical treatment of Angle class II malocclusions patients, the first bicuspid teeth are often needed to be extracted, and all or most of the maxillary extraction spaces need to be provided to the anterior teeth to release the protrusion, so strong anchorage is required for molar anchorage,^[16]^ Traditional anchorage can no longer meet the clinical needs of patients and doctors, and the emergence of micro-implant anchorage has also exposed the deficiency of traditional anchorage.

Micro-implant anchorage is a commonly used orthodontic scheme in clinical orthodontic treatment at present, which is first applied by Kanomi in orthodontic clinical treatment.^[19]^ Through the bone as the direct receiver of the Anchorage force, the reaction force of the orthodontic force is applied on the jaw to avoid unnecessary tooth movement.^[20]^ Micro-implant Anchorage is often used in clinical treatment such as closing the extraction space, depressing the elongation of molars, correcting deep overjet and so on. Micro-implant anchorage is widely used in the treatment of class II malocclusions because of its strong stability, small size, simple operation, and short course of treatment. Micro-implant anchorage correction of class II malocclusions can achieve the following results:

1. Push maxillary molars or maxillary arch backward^[22]^;
2. Recycle the upper anterior teeth and reduce the overlay.
  In order to improve the protrusion type of class II patients, tooth extraction is one of the commonly used methods. The micro-implant anchorage is used to close the extraction space and is often implanted between the maxillary second premolars and the first molars. The anterior teeth are recovered by the contractile force provided by the elastic skin chain or spiral spring mounted on the micro-implants to reduce the coverage and improve the facial shape.^[23]^
3. Depress the anterior teeth and molars.

After micro-implant implantation, the alveolar area can depress the overdeveloped alveolar bone, which is beneficial to the correction of patients with high angle or gingival smile in Angle Class II malocclusions.^[24]^

It is proved clinically that the anti-correction effect of micro-implant anchorage is reliable in the treatment of Angle class II malocclusions. However, the evidence from RCTs is not consistent. With the increasing number of clinical trials, it is urgent to systematically evaluate the efficacy and safety of micro-implant anchorage in the treatment of class II malocclusions. In this study, we will summarize the latest evidence of the efficacy of micro-implant anchorage in the treatment of class II malocclusions. This work also provides useful evidence to determine whether micro-implant anchorage is effective and safe in the treatment of class II malocclusions, which is beneficial to clinical practice and health-related decision-makers.

However, this systematic review has some limitations. There may be some clinical heterogeneity due to the different types and locations of implants, and different degrees of illness and treatment time of patients. Due to the limitation of language ability, we only search English and Chinese literature and may ignore studies or reports in other languages.

## Author contributions

**Data curation:** Kaiting Wang, Hongliang Fan.

**Funding acquisition:** Weihong Xie.

**Resources:** Hongmei Yang, Jianbin Li.

**Software:** Jianbin Li.

**Supervision:** Hongmei Yang.

**Writing – original draft:** Kaiting Wang, Hongliang Fan.

**Writing – review & editing:** Weihong Xie.
